# CD38 and the mitochondrial calcium uniporter contribute to age-related hematopoietic stem cell dysfunction

**DOI:** 10.1097/IN9.0000000000000048

**Published:** 2024-10-08

**Authors:** Connor S. R. Jankowski, Thomas Weichhart

**Affiliations:** 1 Department of Medical Genetics, Center for Pathobiochemistry and Genetics, Medical University of Vienna, Vienna, Austria

**Keywords:** CD38, mitochondrial calcium uniporter, hematopoiesis, aging, nicotinamide adenine dinucleotide metabolism, mitochondria

## Abstract

Hematopoietic stem cells (HSCs) are the multipotent progenitors of all immune cells. During aging, their regenerative capacity decreases for reasons that are not well understood. Recently, Song et al investigated the roles of two metabolic proteins in age-related HSC dysfunction: CD38 (a membrane-bound NADase) and the mitochondrial calcium uniporter that transports calcium into the mitochondrial matrix. They found that the interplay between these proteins is deranged in aged HSCs, contributing to their diminished renewal capacity. These findings implicate compromised nicotinamide adenine dinucleotide metabolism as underlying HSC dysfunction in aging.

Hematopoietic stem cells (HSCs) reside within the bone marrow and give rise to all types of blood cells throughout the mammalian lifespan ^[1]^. However, their ability to self-renew declines with age. Indeed, stem cell exhaustion was established as one of the original hallmarks of aging more than a decade ago ^[2]^. HSCs from older individuals have a reduced proliferative capacity compared to those from young donors ^[3,4]^, and aged HSCs produce reduced lymphoid and enhanced myeloid cell populations ^[4–6]^. Many of the factors that contribute to these phenotypes are considered largely cell intrinsic, although the roles of environmental factors are also recognized ^[4]^.

Several mechanisms contribute to HSC aging, including DNA damage, senescence, epigenetic reprogramming, and impaired mitochondrial activity ^[3,4,6]^. One factor uniting these processes is the small-molecule metabolite nicotinamide adenine dinucleotide (NAD), which decreases with age ^[7]^. NAD is an essential co-factor in a wide array of metabolic reactions, including the tricarboxylic acid (TCA) cycle and oxidative phosphorylation. In addition to its critical role in cellular metabolism, NAD is a substrate for the DNA-repairing enzyme poly-ADP-ribose polymerase, the sirtuin (SIRT) family of proteins, and CD38. Thus, multiple mechanisms implicated in aged HSC dysfunction relate to disruptions to NAD homeostasis.

CD38 is a membrane-bound NADase that converts NAD to cyclic ADP-ribose (cADPR) and ADP-ribose (ADPR) ^[8]^. It has long been thought to play a role in HSC regeneration ^[9,10]^. Expression of CD38 increases with age, and this increase has been implicated as the primary cause of decreased cellular NAD levels ^[11,12]^. CD38 is believed to promote HSC quiescence by regulating calcium signaling, a role it plays in several other immunological contexts ^[13,14]^. The CD38 products cADPR and nicotinic acid adenine dinucleotide phosphate (NAADP) play a role in calcium signaling by promoting the release of calcium stores into the cytoplasm ^[15]^.

Mitochondria are relatively abundant in HSCs and play a major role in buffering changes in cytosolic calcium, primarily by taking up calcium into the matrix through the mitochondrial calcium uniporter (MCU) ^[16,17]^. The subsequent increase in matrix calcium levels is involved in multiple processes depending on cell type, including activation of TCA cycle enzymes ^[16,18,19]^. As such, mitochondrial calcium homeostasis is a multifaceted regulator of HSC function ^[20]^. A calcium-dependent increase in mitochondrial membrane potential precedes HSC cell division ^[21]^, and calcium concentration also appears to influence HSC population phenotypes ^[22]^.

In a recent study published in *Nature Aging*, Song et al ^[23]^ identified that the relationship between NAD, CD38, and MCU was disrupted in HSCs from aged mice. Using a CD38 knock-out (KO) mouse, they show that a lack of CD38 expression hinders cytokine-induced proliferation and that transplanted CD38 KO HSCs demonstrated reduced proliferative capacity in young mice. In line with this observation, HSCs overexpressing CD38 showed an enhanced capacity to reconstitute blood immune cells in lethally irradiated recipient mice. In contrast to these HSC-stimulated proliferation models, HSCs of young CD38 KO mice in their quiescent state under homeostasis showed comparable proliferative potential compared to wild-type (WT). These observations collectively implicate CD38 in mounting a proliferative response in HSCs.

Through RNA-Seq analysis of cytokine-stimulated WT and CD38 KO HSCs, Song et al identified clusters of differentially expressed cell cycle and mitochondrial genes. Importantly, there was again no difference in the expression of these genes between the two genotypes under quiescent conditions. Stable isotope tracing using [U-^13^C]-glucose demonstrated a functional mitochondrial deficit in stimulated CD38 KO HSCs, as these cells showed decreased labeling into the TCA intermediates succinate and fumarate. Complementary metabolomics data also suggested that CD38 KO HSCs had an increased reliance on glycolysis, as these cells had higher levels of the glycolytic intermediates fructose 1,6-bisphosphate and lactate. These cells also showed decreased ATP levels. Together, these observations indicate that CD38 supports mitochondrial integrity in cytokine-stimulated HSCs in young mice.

The authors then found that HSCs from CD38 KO mice had reduced levels of cytosolic and mitochondrial calcium compared to those from WT mice. Mitochondria in these cells had a decreased membrane potential and superoxide load. In WT mice, cellular CD38 expression level was correlated with membrane potential and the levels of reactive oxygen species (ROS). To further examine the role of mitochondrial calcium uptake in this paradigm, they generated Vav-iCre^+/−^;MCU^loxP/loxP^ mice, deleting the MCU from HSCs. Like the HSCs from CD38 KO mice, these MCU KO HSCs displayed similar characteristics under quiescent conditions but reduced engraftment and proliferative capacity following bone marrow transplantation. These cells also showed reduced calcium signaling, mitochondrial membrane potential, and mitochondrial superoxide. MCU shRNA treatment in CD38 KO HSCs improved their calcium levels and colony-forming ability, suggesting that regulation of HSC function by CD38 involves mitochondrial calcium signaling via MCU.

Next, they examined this system in HSCs from aged mice. Concomitant with decreased NAD levels, HSCs from aged mice showed increased CD38 expression along with elevated calcium signaling and mitochondrial stress. CD38 expression has already previously been shown to increase with age in other immune and tissue cells ^[11,12]^. Interestingly, HSCs from aged CD38 KO mice had elevated NAD levels compared to those from aged WT mice, and aged CD38 KO mice also had comparatively higher white blood cell and lymphocyte counts. The lack of CD38 also alleviated myeloid-biased differentiation in peripheral blood. Compared to HSCs from aged WT mice, HSCs from aged CD38 KO mice also performed better in transplantation engraftments. Similarly, in aged mice with MCU KO HSCs, white blood cell and lymphoid cell counts were increased, and blood marrow cellularity was improved. These HSCs performed better than aged WT HSCs in competitive transplantation assays. Impressively, improved performance of HSCs from aged CD38 KO mice did not appear to depend on a lifelong lack of CD38 activity, as treatment with a CD38 inhibitor recapitulated these effects in aged WT mice. Thus, restricting CD38 activity in aged mice helps rejuvenate and restore HSC integrity.

This illuminating new work by Song et al identifies CD38 as a regulator of proliferation in stimulated HSCs and connects CD38 activity in HSCs with mitochondrial calcium uptake via MCU. They further establish that dysregulation of CD38 and MCU dynamics in aged mice contributes to HSC dysfunction. These results are especially interesting because a small-molecule CD38 inhibitor improved the HSC phenotype in aged mice, suggesting that the window for HSC therapy may remain open late in life. This study highlights CD38 and MCU as potential therapeutic targets and contributes to a growing body of literature demonstrating the potential for modulation of NAD metabolism to improve HSC function in aged individuals. In this study, CD38 inhibition by the small-molecule inhibitor 78c decreased myeloid-biased HSCs in the bone marrow of aged mice, and depletion of myeloid-biased HSCs in aged mice produces a more youthful immune system ^[24]^. Overexpression of SIRT3 in aged HSCs improves their regenerative capacity ^[25]^, and treatment with the NAD precursor nicotinamide riboside promotes a youthful metabolic phenotype in HSCs from aged mice ^[26]^. Together, these studies show the power of immunometabolism to guide therapeutic discovery and development.

## Conflicts of interest

The authors declare that they have no conflicts of interest.

## Funding

Research in the Weichhart Lab is supported by funding from the following sources: the Austrian Science Fund (FWF) grants P34023-B, P34266-B, FWF Sonderforschungsbereich F8301, F8308, and the Ann Theodore Foundation Breakthrough Sarcoidosis Initiative.
